# Comparative Characterization of Olfactory Dysfunction in Mouse Models of Eosinophilic Chronic Rhinosinusitis

**DOI:** 10.3390/cells15121118

**Published:** 2026-06-20

**Authors:** Agnès Dekeyser, Marylène Lecocq, Alessandra Camboni, Sophie Gohy, Charles Pilette, Brian Lin, Caroline Huart, Valérie Hox

**Affiliations:** 1 Pole of Lung, Nose and Skin Research (LUNS), Institute of Experimental and Clinical Research (IREC), UCLouvain, 1200 Brussels, Belgium; agnes.dekeyser@uclouvain.be (A.D.); marylene.lecocq@uclouvain.be (M.L.); sophie.gohy@uclouvain.be (S.G.); charles.pilette@saintluc.uclouvain.be (C.P.); 2 Laboratory of Gynecology, Institute of Experimental and Clinical Research (IREC), UCLouvain, 1200 Brussels, Belgium; alessandra.camboni@saintluc.uclouvain.be; 3 Department of Pathology, Cliniques Universitaires Saint-Luc, 1200 Brussels, Belgium; 4 Department of Pulmonology, Cliniques Universitaires Saint-Luc, 1200 Brussels, Belgium; 5 Department of Developmental, Molecular and Chemical Biology, Tufts University Graduate School of Biomedical Sciences, Boston, MA 02111, USA; brian.lin@tufts.edu; 6 Institute of Neurosciences, UCLouvain, 1200 Brussels, Belgium; caroline.huart@saintluc.uclouvain.be; 7 Department of Otorhinolaryngology, Head and Neck Surgery, Cliniques Universitaires Saint-Luc, 1200 Brussels, Belgium

**Keywords:** eosinophilic chronic rhinosinusitis, olfactory dysfunction, mouse model, inflammation, olfactory sensory neurons

## Abstract

Eosinophilic chronic rhinosinusitis (eCRS) is an upper respiratory condition frequently associated with olfactory dysfunction (OD). Despite its high prevalence, the mechanisms underlying OD remain poorly understood. Several eCRS models have been described, but their olfactory phenotypes are poorly characterized. In this study, we compared two of the most frequently used mouse models of eCRS in order to standardize in vivo research on eCRS-related OD. Male and female mice were challenged with ovalbumin (OVA) combined with *Staphylococcus aureus* enterotoxin B (SEB) over a 13-week protocol or with OVA combined with *Aspergillus oryzae* protease (AP) for 6 or 12 weeks. Olfactory function was assessed using the buried food test and habituation/dishabituation test. After sacrifice, the integrity and inflammation of the olfactory epithelium were assessed on coronal skull sections by (immuno)histology, including sex as a biological variable. Both models exhibited impaired olfactory function, reduced olfactory epithelium surface area and thickness, and eosinophil infiltration of the olfactory mucosa. The OVA-AP model showed additional presence of neutrophils in the olfactory mucosa, suggesting a mixed inflammatory response. No functional or histological difference was detected between male and female mice, except for epithelial thickness in OVA-SEB control mice. Overall, both murine models are suitable for mechanistic studies of OD in eCRS, with the AP-12-week model displaying the most pronounced inflammation.

## 1. Introduction

Olfaction plays a fundamental role in daily life, contributing to hazard detection, food intake, and social behavior. Olfactory dysfunction (OD), defined as a partial or complete loss of smell, has long been underrecognized and gained broader attention after the SARS-CoV-2 pandemic in 2019. OD is associated with substantial impairments in quality of life, including reduced appetite, compromised safety, altered social interactions, and decreased work performance [1,2].

Beyond aging, sinonasal diseases represent the leading cause of OD, accounting for approximately 67% of cases, among which we find chronic rhinosinusitis (CRS) [3]. CRS is a frequent chronic inflammatory disease of the nasal and paranasal sinus mucosa, affecting around 11% of the Western population [4]. It is defined by persistent symptoms such as nasal obstruction, rhinorrhea, facial pressure, and reduced or lost olfaction, lasting longer than 12 weeks. CRS is classified into two main clinical phenotypes: CRS with nasal polyps (CRSwNP), commonly associated with type 2 (T2) inflammation with eosinophilic infiltration, and CRS without nasal polyps (CRSsNP), more frequently linked to a non-type-2 inflammatory profile with neutrophilic infiltration [5]. OD is known to be more severe in patients with CRSwNP than in those with CRSsNP [2].

CRS-associated OD is believed to arise from multiple, often overlapping mechanisms. Conductive factors, such as obstruction of the nasal cavity by mucosal edema or nasal polyps, impair airflow and limit the delivery of odorants to the olfactory cleft. In parallel, sensorineural mechanisms, including inflammation-mediated injury to the olfactory epithelium and downstream neural pathways, contribute to olfactory impairment. Additional processes, such as mucus alteration, epithelial remodeling, microbial dysbiosis, and structural or functional alterations in olfactory-related brain regions, have been put forward as potential contributors [2,5,6].

Despite advances in medical and surgical management of CRS, OD remains one of the most persistent and difficult-to-treat symptoms. A deeper understanding of CRS-induced OD is essential to optimize treatment strategies, elucidate recovery mechanisms, and develop more effective, cost-efficient therapies for affected patients [6].

Mechanistic investigation of OD in CRS is limited in humans, largely due to restricted access to olfactory tissue. These limitations highlight the value of murine models, which offer a well-characterized olfactory anatomy in a sinonasal system that is not very different from humans, and allow controlled induction of CRS, enabling detailed analysis of inflammatory processes, tissue remodeling, and olfactory outcomes.

Several murine models have been developed to study CRS and its associated inflammatory processes, using diverse strategies such as allergen exposure [7], administration of bacterial [8,9] or fungal [10,11] components, and genetic manipulations [12]. Although these models have failed to reproduce the human presentation of nasal polyps, some of them represent an adequate image of type 2 eosinophilic CRS (eCRS) [13] and this absence of polyps even allows for focus on the inflammatory cause of OD. However, their heterogeneity, combined with the limited assessment of OD, has resulted in the use of non-comparable models. Consequently, there is currently no consensus on a murine model that reliably recapitulates both chronic sinonasal inflammation and robust, quantifiable OD comparable to the human condition.

To address this gap, we conducted a comparative evaluation of the two most frequently reported murine models of eCRS using either the experimental ovalbumin (OVA) allergen in combination with *Staphylococcus aureus* enterotoxin B (SEB) or OVA with *Aspergillus oryzae* protease (AP). OD-related features of these models were compared across key domains, including anatomical regions of alterations, olfactory epithelial changes and inflammatory profiles, whilst considering gender-related differences. The objective of this study was to identify potential differences between models and validate the most relevant murine model for investigating eCRS-associated OD in future experimental and translational studies.

## 2. Materials and Methods

### 2.1. Animals

Five-week-old male and female BALB/cAnNRj mice were purchased from Janvier Labs (Le Genest-Saint-Isle, France) and housed in an animal facility maintained at 22–25 °C and 50–60% relative humidity. BALB/c mice were selected because previous research has shown that this strain is prone to induce stronger T2 inflammatory responses [14,15]. All animal experiments were performed in compliance with the ethical committee guidelines, Commission d’Ethique relative à l’Expérimentation Animale du Secteur des Sciences de la Santé, UCLouvain, and approved under reference code UCL/MD/2024/65 on 14 October 2024. All experiments were performed within a single cohort of animals processed under identical experimental conditions. No predefined inclusion or exclusion criteria were established for animals or data points, and no exclusions were made. Animals were allocated to control and CRS groups by random assignment. To minimize potential confounders, the order of group testing and the order of handling within each group were randomized. Investigators were aware of group allocation during the conduct of the experiment but were blinded to group identity during outcome assessment and data analysis through recording of samples.

### 2.2. Experimental Protocol

**Figure 1:**
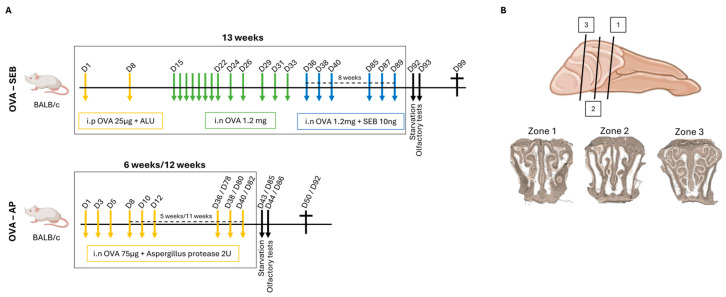
Experimental protocol for eCRS induction in two murine models. (**A**) Chronic rhinosinusitis induction in two mouse models, one for 13 weeks and the other for either 6 or 12 weeks, as described before [13,16]. (**B**) Coronal skull sections for histological analysis were analyzed at three different regions of the olfactory cleft. i.n.: intranasal; i.p.: intraperitoneal; OVA: ovalbumin; ALU: aluminum hydroxide gel; SEB: *Staphylococcus aureus* enterotoxin B; AP: *Aspergillus oryzae* protease.

After a one-week acclimatization period, mice were divided into two models of eCRS: ovalbumin (OVA)–*Staphylococcus aureus* enterotoxin B (SEB) or OVA–*Aspergillus oryzae* protease (AP) (Figure 1A).

OVA-SEB model. Mice (4 per sex) were sensitized with intraperitoneal (i.p.) injection of 25 µg OVA (Grade V, Sigma-Aldrich, St. Louis, MO, USA) in 2 mg of aluminum hydroxide gel (vac-alu-50, InvivoGen, San Diego, CA, USA) in a total volume of 200 μL on days 1 and 8. This was followed by intranasal (i.n.) instillation of 1.2 mg OVA in a total volume of 20 µL per administration. Mice first received OVA daily for one week, and then three times per week for two additional weeks. Subsequently, i.n. instillation of 1.2 mg OVA (20 µL) was combined with SEB (10 ng in 20 µL, S4881, Sigma-Aldrich, St. Louis, MO, USA) and administered three times per week for 8 weeks. Control mice (CTRL; 4 per sex) received Phosphate-Buffered Saline (PBS) according to the same administration route and schedule.

OVA-AP model. Mice (4 per sex/time point) were sensitized with i.n. instillation of 75 µg OVA in combination with 2U AP (P6110, Sigma-Aldrich, St. Louis, MO, USA) in a total volume of 20 µL, 3 times a week for either 6 or 12 weeks. Control mice (4 per sex/time point) received PBS according to the same administration route and schedule.

### 2.3. Olfactory Testing

Four days after the instillation period, two olfactory tests were performed, namely the buried food test and the habituation/dishabituation test.

For the buried food test that studies time to odor detection in mice, animals were food-deprived for 20 h prior to testing. Each mouse was first placed individually in a clean cage containing fresh bedding for 10 min for habituation, and then transferred to a second clean cage in which a food pellet was buried approximately 1 cm beneath the bedding surface. To account for exploratory behavior, the food pellet was positioned away from the cage corners. The latency to locate the food was recorded, with a maximum test duration of 5 min.

The habituation/dishabituation test was used to assess the ability of mice to discriminate between different odorants. Mice were placed individually in a clean cage for a 5 min acclimation period. Cotton swabs impregnated with odorants were then presented sequentially for 2 min each, with a 1 min interstimulus interval. The sequence consisted of five presentations of water (neutral control), followed by six presentations of geraniol and six presentations of lime. These odorants have been previously used in olfactory experiments [17,18]. Sniffing behavior was video-recorded and quantified offline. Mice with intact olfactory function exhibit a progressive decrease in sniffing duration upon repeated exposure to the same odorant (habituation) and an increase in sniffing when a novel odorant is introduced (dishabituation).

### 2.4. Sacrifice and Tissue Sampling

Following olfactory testing, mice were euthanized with an i.p. injection of pentobarbital (200 mg/kg). Mice were subsequently decapitated and the skin covering the head was removed. Skulls were fixed in formol for 24 h, rinsed overnight in saline solution with gentle agitation, and decalcified in OsteoRAL R (RAL Diagnostics, Martillac, France) for 5 days. After a final overnight wash, skulls were embedded in paraffin according to standard procedures. Paraffin blocks were sectioned coronally at either 3 or 5 µm thickness at three defined depths of the nasal cavity for histological analysis. The three zones were defined based on the ethmoid turbinates present at each level. Zone 1 corresponds to the appearance of the first ethmoturbinates. In Zone 2, the fourth ethmoturbinate becomes visible, while in Zone 3, the fifth and sixth ethmoturbinates begin to appear (Figure 1B). Slides were deparaffinized and rehydrated through successive toluene and methanol baths for subsequent analyses.

### 2.5. Histological Analyses

Tissue sections were stained with hematoxylin and eosin (H&E) to assess epithelial thickness and eosinophilic infiltration. Epithelial thickness was measured from the basal membrane to the top of the olfactory knobs, on both sides of the nasal septum, 550 µm under the top of the nasal cavity, using QuPath software version 0.5.1 [19]. Eosinophilic infiltration was assessed by counting the number of eosinophils in three defined regions (0.035 mm^2^ each) along both sides of the nasal septum, located 300 µm, 800 µm, and 1300 µm from the top of the nasal cavity. Counts from all regions were averaged per animal to obtain a single value for statistical analysis.

### 2.6. Immunohistochemistry

Immunohistochemistry was performed to assess the proportion of olfactory epithelium (OE) defined by the presence of olfactory sensory neurons (OSNs) within the nasal cavity and to evaluate neutrophilic infiltration. Following antigen retrieval in citrate buffer and blocking, sections were incubated with primary antibodies for 1 h. The primary antibodies used were anti-olfactory marker protein (OMP; 1:500, sc-365818, Santa Cruz Biotechnology, Dallas, TX, USA) for OSNs and anti-Ly6G (1:2000, 551459, BD Pharmingen, San Diego, CA, USA) for neutrophils. For Ly6G staining, an additional 1 h incubation with rabbit anti-rat IgG (H + L) secondary antibody (1:100, AI-4001, Vector Laboratories, Newark, CA, USA) was performed. Sections were then incubated with poly-HRP-conjugated secondary antibodies (goat anti-mouse IgG, #B40961; goat anti-rabbit IgG, #B40962; Invitrogen, Carlsbad, CA, USA) for 40 min. Signal detection was performed using the Liquid DAB+ Substrate Chromogen System (K3468, Dako, Glostrup, Denmark) according to the manufacturer’s instructions. Sections were counterstained with Hemalum for 5 min, mounted, and scanned using a Pannoramic Scan II system (3DHistech, Budapest, Hungary). Quantitative analysis was performed using QuPath software version 0.5.1 [19]. OE proportion was quantified using a thresholding method and normalized to the total nasal cavity area. The threshold settings were applied to all slides to ensure consistency across samples. Neutrophilic infiltration was assessed by counting Ly6G-positive cells in three defined regions (0.035 mm^2^ each) along both sides of the nasal septum, located 300 µm, 800 µm, and 1300 µm from the top of the nasal cavity. Counts from all regions were averaged per animal to obtain a single value for statistical analysis.

### 2.7. Statistical Analysis

Statistical analyses were conducted with GraphPad Prism version 8.0.2 (GraphPad Software, Boston, MA, USA). Normality was assessed using the Shapiro–Wilk test. Data were analyzed using two-way ANOVA with sex (female, male) and group (CTRL, eCRS) as fixed factors to assess main effects and their interaction. When no significant sex effect or sex × group interaction was detected, data from males and females were pooled for subsequent analyses. For comparisons across experimental models, a separate two-way ANOVA with group and model as factors was performed. Post hoc multiple comparisons were conducted using the Tukey test when ANOVA revealed significant main effects or interactions. Pearson correlation was used to assess the relationship between buried food test scores and OMP-positive area, epithelial thickness, or inflammatory infiltration. For the olfactory habituation/dishabituation test, sniffing times were analyzed using two-way repeated-measures ANOVA to compare presentation times within each odor, and t-tests were used to compare sniffing times between consecutive odors. All data are presented as mean ± standard deviation (SD), and *p* < 0.05 was considered statistically significant.

## 3. Results

### 3.1. eCRS-Related Findings Are Not Influenced by Gender of Mice

All datasets were initially analyzed using two-way ANOVA with treatment and sex as factors to determine whether group differences varied between males and females. For most parameters, no significant effect of sex or sex × treatment interaction was detected. A significant sex effect and interaction were only observed for epithelial thickness in the OVA-SEB group, with differences restricted to females. Given the absence of sex effects across the remaining analyses, data from males and females were combined for presentation in the main figures, while sex-stratified analyses are provided in the Supplementary Data (Figure S1).

### 3.2. OVA-SEB and OVA-AP Models Exhibited Reduced Odor Detection in the Buried Food Test Without Affecting Habituation/Dishabituation Responses

Olfactory performance was assessed using two behavioral assays: the buried food test, which measures the ability to locate a hidden food pellet, and the habituation/dishabituation test, which evaluates odor detection and discrimination.

**Figure 2:**
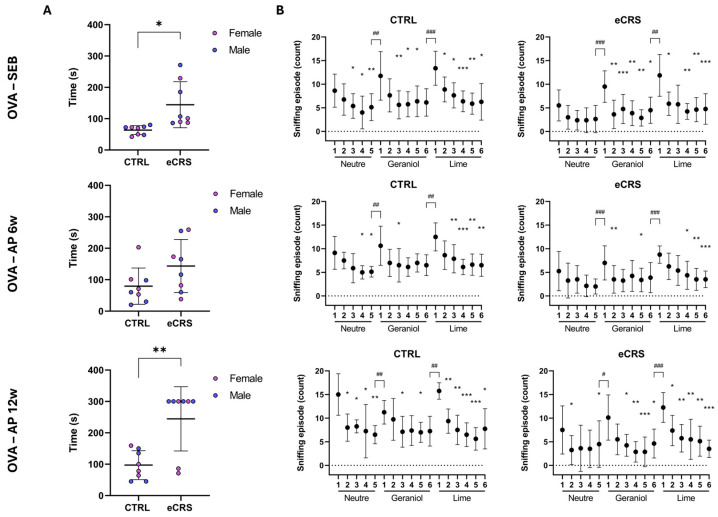
OVA-SEB and OVA-AP treatments impair odor detection in the buried food test without affecting habituation/dishabituation responses. (**A**) Buried food test. Time in seconds to retrieve a buried food pellet is shown according to sex (Female and Male) and group (eCRS and CTRL), in the two mouse models studied. (**B**) Habituation/dishabituation test. Representation of the ability of mice to recognize an odor (habituation: *) and discriminate between two odors (dishabituation: #), measured by the sniffing time at each odor presentation, in each group and mouse model (n = 4/sex/group/model). Data are expressed as mean ± SD. Two-way ANOVA (**A**): * *p* < 0.05; ** *p* < 0.01. Two-way repeated-measures ANOVA (**B**: habituation): * *p* < 0.05; ** *p* < 0.01; *** *p* < 0.001. T-test (**B**: dishabituation): # *p* < 0.05; ## *p* < 0.01; ### *p* < 0.001. CTRL: control; eCRS: eosinophilic chronic rhinosinusitis.

In the buried food test, eCRS mice consistently required more time to locate the buried food pellet than CTRL mice across all models, despite comparable exploratory behavior within the testing arena. Specifically, significant increases in search time were observed in eCRS mice in the OVA-SEB model (*p* < 0.05, *ηp*^2^ = 0.42, 95% CI [0.02,0.66]) and OVA-AP 12-week models (*p* < 0.01, *ηp*^2^ = 0.58, 95% CI [0.13,0.75]), while the OVA-AP 6-week model showed a similar, non-significant trend (*p* = 0.1116, *ηp*^2^ = 0.20, 95% CI [0,0.50]) (Figure 2A). When comparing the two models, we found a significant effect of group (*p* < 0.001) and model (*p* = 0.0172), with no significant group × model interaction (*p* = 0.2073), suggesting that the magnitude of olfactory impairment was consistent between the OVA-SEB and OVA-AP models. Tukey’s post hoc comparison confirmed a significant difference between OVA-SEB and OVA-AP 12w (p_adj = 0.0243) (Figure S2A).

Even though eCRS mice showed a lower sniffing time compared to CTRL mice, habituation/dishabituation analyses showed no difference between CTRL and eCRS mice at the level of habituation to repeated odorant presentations and dishabituation upon presentation of a novel odorant for any of the tested models (Figure 2B). In the OVA-SEB model, CTRL and eCRS mice showed significant habituation and dishabituation for most odorants, with the exception of habituation to the neutral odor (water) in eCRS mice. In the OVA-AP models, CTRL mice exhibited significant habituation and dishabituation for most odorants, although statistical significance was less consistent for geraniol. eCRS mice displayed similar patterns of habituation and dishabituation, except for the neutral odor.

### 3.3. Comparison of Three Nasal Zones Reveals the Highest Abundance of Olfactory Neurons at the Most Posterior Coronal Level

**Figure 3:**
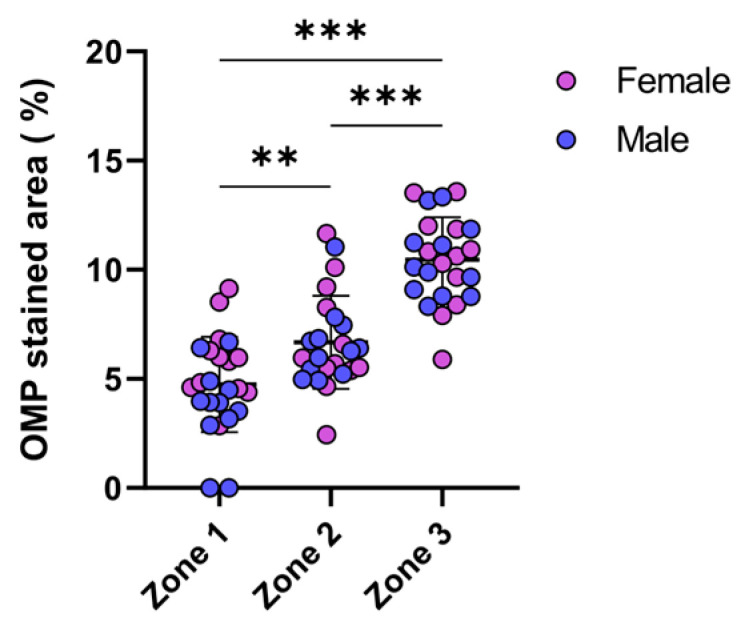
Comparison of three nasal zones shows the highest abundance of olfactory neurons in the most posterior coronal section. Olfactory epithelium presence as defined by the area positive for the olfactory marker protein (OMP), specific to neurons, in three zones of the nasal cavity, with sex and model combined. Data are expressed as mean ± SD. Two-way ANOVA and Tukey test: ** *p* < 0.01; *** *p* < 0.001.

We analyzed olfactory marker protein (OMP) staining at three distinct coronal levels of the nasal mucosa to localize the olfactory epithelium and identify the region in which it is most abundant. Control mice of both models were combined for analysis. OMP staining was significantly higher in Zone 3 compared with Zones 1 and 2 (*p* < 0.001) (Figure 3). Based on these results, Zone 3 was selected for all subsequent analyses.

### 3.4. Mature Olfactory Sensory Neurons Are Reduced in Both OVA-SEB- and OVA-AP-Treated Mice

**Figure 4:**
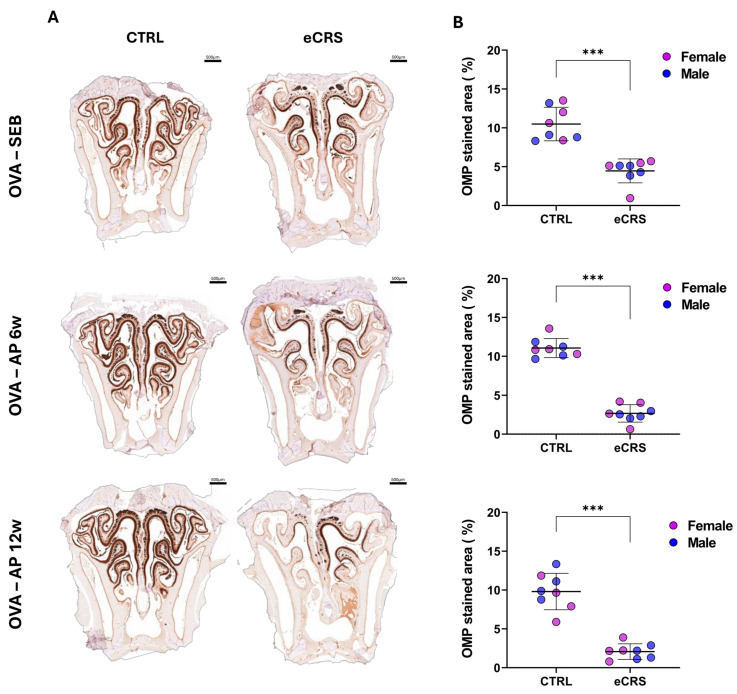
Mature olfactory sensory neurons are reduced in OVA-SEB- and OVA-AP-treated mice. (**A**) Coronal tissue slides of the olfactory mucosa with staining of the olfactory marker protein (OMP), specific to neurons, by immunohistochemistry in the two mouse models. Sections are representative samples randomly selected from each model and group. Scale bar: 500 µm. (**B**) Quantification of OMP+ area by sex (Female and Male) and groups (CTRL and eCRS) in the two mouse models studied. Data are expressed as mean ± SD. Two-way ANOVA: *** *p* < 0.001. CTRL: control; eCRS: eosinophilic chronic rhinosinusitis.

Histological sections were immunolabeled for OMP to visualize olfactory sensory neurons in each model. Qualitative inspection of the slides revealed reduced OMP staining in eCRS groups, particularly at the base of the nasal septum and along the turbinates (Figure 4A). This observation was confirmed by quantitative analysis, which revealed a significant reduction in OMP-positive area in eCRS mice compared with their respective controls in all models (*p* < 0.001, OVA-SEB: *ηp*^2^ = 0.76, 95% CI [0.39,0.86]; OVA-AP 6w: *ηp*^2^ = 0.94, 95% CI [0.82,0.96]; OVA-AP 12w: *ηp*^2^ = 0.86, 95% CI [0.61,0.92]) (Figure 4B). Correlation analysis between OMP-positive area and buried food test performance revealed no significant relationship in any model, with R^2^ values ranging from 0.01 to 0.10 and all *p*-values exceeding 0.4 (Figure S3A). Across models, eCRS consistently reduced OE area (*p* < 0.001), with a significant effect of model (*p* = 0.0392) and no significant group × model interaction (*p* = 0.1268), indicating that the effect of eCRS was consistent between the OVA-SEB and OVA-AP models. Tukey’s post hoc comparison confirmed a significant difference between OVA-SEB and OVA-AP 12w (p_adj = 0.0314) (Figure S2B).

### 3.5. Olfactory Epithelium Thickness Is Reduced in OVA-SEB- and OVA-AP-Treated Mice

**Figure 5:**
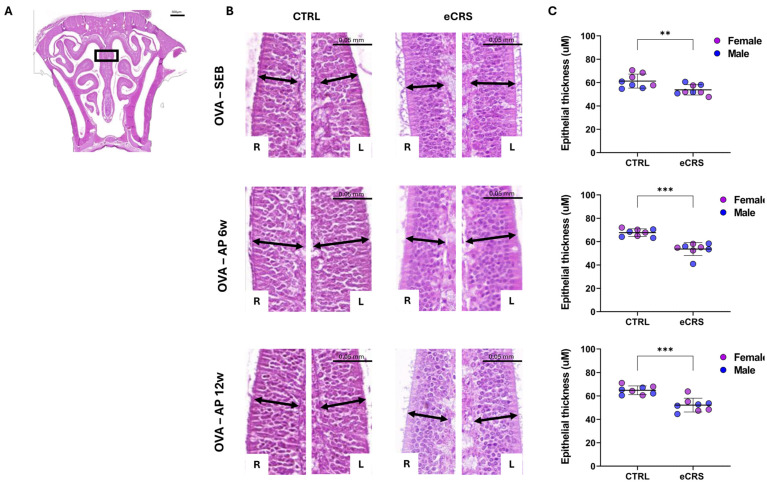
Olfactory epithelium thickness is reduced in OVA-SEB- and OVA-AP-treated mice. (**A**) Representative histological section randomly selected from a control mouse illustrating the region of the nasal septum (black box) used for epithelial thickness measurements. Both sides were averaged for quantification. Scale bar: 500 µm. (**B**) Higher magnification of both sides of the analyzed nasal septum region. Sections are representative samples randomly selected from each model and group. Double-headed arrows indicate epithelial thickness measurements. Scale bar: 50 µm. (**C**) Quantification of epithelial thickness according to sex and group in each mouse model. Data are expressed as mean ± SD. Two-way ANOVA: ** *p* < 0.01; *** *p* < 0.001. CTRL: control; eCRS: eosinophilic chronic rhinosinusitis.

Epithelial thickness was measured in the superior region of the nasal septum on both sides and pooled for analysis (Figure 5A). This region was selected because OE seems to be maintained in most cases, including in eCRS mice, whereas other regions, such as the inferior septum, frequently lose their olfactory epithelium, as demonstrated in Figure 4. Although qualitative differences were not readily apparent on histological sections (Figure 5B), quantitative analysis revealed a significant reduction in epithelial thickness in eCRS mice compared with CTRL mice in both models (OVA-SEB: *p* < 0.01, *ηp*^2^ = 0.33, 95% CI [0.07,0.53]; OVA-AP 6w: *p* < 0.001, *ηp*^2^ = 0.52, 95% CI [0.24,0.68]; OVA-AP 12w: *p* < 0.001, *ηp*^2^ = 0.49, 95% CI [0.21,0.66]) (Figure 5C). Two-way ANOVA confirmed a main effect of eCRS (*p* < 0.001), with no significant effect of model (*p* = 0.1734) or interaction (*p* = 1282), showing that eCRS consistently reduced epithelial thickness across models (Figure S2C).

### 3.6. OVA-AP-Treated Mice Show More Pronounced Eosinophilic Infiltration in the Olfactory Epithelium than OVA-SEB-Treated Mice

**Figure 6:**
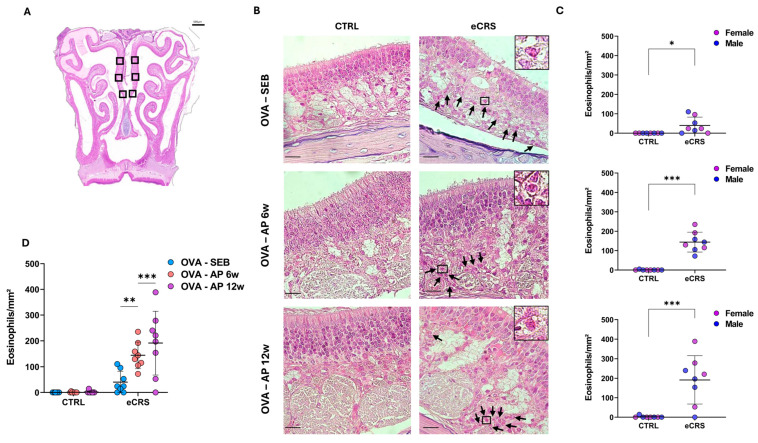
Eosinophilic infiltration is enhanced in OVA-AP-treated mice compared with OVA-SEB-treated mice. (**A**) Representative histological section randomly selected from an eCRS mice showing the six 0.035 mm^2^ regions (black boxes) analyzed for eosinophil counts. Counts were averaged per animal for quantification. Scale bar: 500 µm. (**B**) Higher magnification of the olfactory mucosa stained for eosinophils by H&E. Sections are representative samples randomly selected from each model and group. Arrows indicate eosinophils. Scale bar: 20 µm. (**C**) Comparison of eosinophil counts between CTRL and eCRS groups in each model. (**D**) Comparison of eosinophil count in each model according to groups (CTRL and eCRS). Data are expressed as mean ± SD. Two-way ANOVA: * *p* < 0.05; ** *p* < 0.01; *** *p* < 0.001. Post hoc multiple comparisons were conducted using Tukey test. CTRL: control; eCRS: eosinophilic chronic rhinosinusitis.

Eosinophils were quantified on H&E-stained sections from the most posterior region of the nasal cavity. Counts were pooled from three distinct areas along both sides of the nasal septum, selected to capture both impaired and intact OE (Figure 6A). Qualitative examination of histological sections revealed increased eosinophil infiltration in eCRS mice compared with CTRL mice across all models (Figure 6B), with eosinophils predominantly localized in the subepithelial region. Quantitative analysis confirmed that eCRS mice exhibited significantly higher eosinophil counts than CTRL mice in each model (OVA-SEB: *p* < 0.05, *ηp*^2^ = 0.34, 95% CI [0, 0.61]; OVA-AP 6-week: *p* < 0.001, *ηp*^2^ = 0.84, 95% CI [0.55,0.91]; OVA-AP 12-week: *p* = 0.001, *ηp*^2^ = 0.60, 95% CI [0.13,0.77]) (Figure 6C). Notably, eosinophil counts in the OVA-AP 12-week eCRS group displayed marked inter-individual variability (39.88 ± 25.77). Correlation analysis between eosinophil counts and buried food test performance focusing on CRS mice only revealed no significant relationship in any model, with R^2^ values ranging from 0.02 to 0.31 and all *p*-values exceeding 0.1 (Figure S3B).

Comparison across models using two-way ANOVA revealed a significant group × model interaction. Post hoc analyses demonstrated that eosinophil infiltration in eCRS mice was significantly greater in the OVA-AP model than in the OVA-SEB model at both 6 weeks (*p* < 0.01) and 12 weeks (*p* < 0.001), indicating a more pronounced eosinophilic inflammatory response in the OVA-AP models (Figure 6D).

### 3.7. OVA-AP-Treated Mice Show an Increased Neutrophilic Infiltration in the Olfactory Epithelium That Is Not Observed in OVA-SEB-Treated Mice

**Figure 7:**
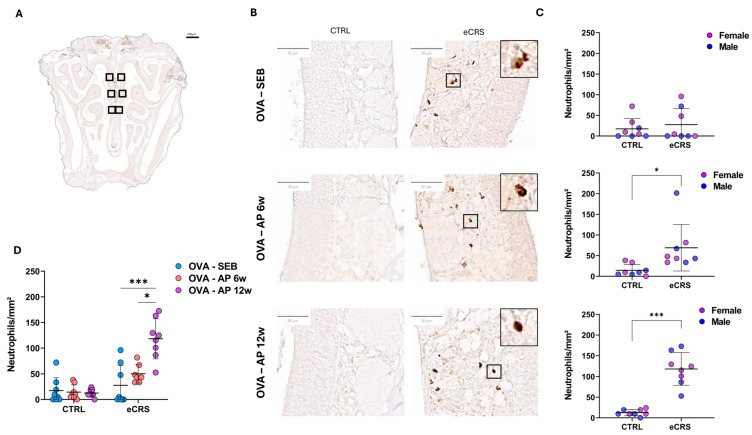
OVA-AP-treated mice exhibit greater neutrophil infiltration in the olfactory epithelium than OVA-SEB-treated mice. (**A**) Representative histological section randomly selected from a control mouse showing the six 0.035 mm^2^ regions analyzed for neutrophil counts. Counts were averaged per animal for quantification. Scale bar: 500 µm. (**B**) Higher magnification of the olfactory mucosa stained for neutrophils by immunohistochemistry. Sections are representative samples randomly selected from each model and group. Scale bar: 50 µm. (**C**) Quantification of neutrophil counts in CTRL and eCRS groups in each model. (**D**) Comparison of neutrophil count in each model according to groups (CTRL and eCRS). Data are expressed as mean ± SD. Two-way ANOVA: * *p* < 0.05; *** *p* < 0.001. Post hoc multiple comparisons were conducted using Tukey test. CTRL: control; eCRS: eosinophilic chronic rhinosinusitis.

Neutrophils were quantified at three distinct areas along the nasal septum from the most posterior region of the nasal cavity on both sides, selected to capture both impaired and intact OE (Figure 7A), and counts were averaged across heights and sides for statistical analysis. Neutrophils were observed in eCRS mice in both models (Figure 7B). Quantitative analysis showed no significant difference between eCRS and CTRL mice in the OVA-SEB model (*p* = 0.5470, *ηp*^2^ = 0.03, 95% CI [0, 0.32]), whereas eCRS mice in the OVA-AP model exhibited significantly higher neutrophil counts than controls, particularly after 12 weeks of instillation (6w: *p* = 0.0220, *ηp*^2^ = 0.61, 95% CI [0.14, 0.77]; 12w: *p* < 0.001, *ηp*^2^ = 0.80, 95% CI [0.47, 0.88]) (Figure 7C). Correlation analysis between neutrophil counts and buried food test performance in CRS mice revealed no significant relationship in any model, with R^2^ values ranging from 0.006 to 0.27 and all *p*-values exceeding 0.1 (Figure S3C).

Two-way ANOVA revealed significant main effects of group and model, as well as a significant group × model interaction, indicating that the magnitude of eCRS-induced inflammation depended on the model. Post hoc comparisons showed that neutrophil counts were highest in the OVA-AP 12-week model, significantly exceeding those in both the OVA-SEB (*p* < 0.001) and OVA-AP 6-week models (*p* = 0.0175) (Figure 7D).

## 4. Discussion

Multiple eCRS mouse models have been described in the literature using diverse experimental approaches. However, some of these models have been reported only once, leading to substantial variability in outcomes and limiting cross-study comparability. Moreover, most studies focused primarily on the respiratory mucosa of the sinonasal cavity, while very few have focused on changes in the olfactory region. Therefore, we aimed to compare the olfactory outcomes of the two most commonly used eCRS mouse models, considering treatment regimens, sex, and inflammatory and histopathological changes, with the goal of identifying an optimal model and standardizing OD research in mice. In this study, we show that both tested models, OVA combined with SEB and OVA combined with AP, at two different timepoints, are effective in inducing OE inflammation and disruption, leading to a similar functional OD in both male and female mice as measured by the buried food test. The 12-week OVA-AP model showed the most pronounced inflammatory changes at the level of the olfactory epithelium including both eosinophil and neutrophil infiltration, while OVA-SEB only mounted an eosinophilic response.

One of the aims of this study was to compare male and female mice since humans present well-known gender-related differences in olfaction. In healthy individuals, women are known to outperform men in olfactory testing [20], while men seem to present more often with OD than women [21]. This might be linked to anatomical, hormonal, and genetic differences. To address this potential difference in mice, we compared female and male mice for the eCRS models that were tested, but none of the parameters showed a difference between the two sexes. These findings suggest that, within the experimental conditions tested, both sexes respond similarly to eCRS induction, supporting the use of both sexes in these models.

As the two mouse models were originally established in different strains [22,23], we implemented both models in a single genetic background to facilitate direct comparison. BALB/c mice were selected because they are known to mount strong immune responses following immunization and to develop pronounced T2 inflammation [24,25].

Both models use the administration of OVA in combination with either SEB [23] or AP [22]. While the precise mechanisms underlying inflammation following these treatments remain under investigation, SEB and AP seem to act as an adjuvant to the allergen OVA by, among others, acting as a superantigenic T-cell amplifier [24] or activating the protease-activated receptor-2 (PAR-2) signaling pathways [25], respectively. So far, no studies have investigated their specific mechanisms in OE inflammation. In this paper, we showed that AP as an adjuvant induced more robust inflammation of the OE than SEB, characterized by greater eosinophil infiltration and an additional neutrophilic influx. A similar inflammatory response to AP has been reported previously [16]. This might be explained by the fact that AP, through its direct proteolytic effects on epithelial tight junctions, likely delivers a strong epithelial “danger” and barrier injury signal that preferentially promotes neutrophil recruitment and activation. In contrast, SEB primarily acts as a superantigenic T-cell amplifier without providing the same protease-mediated innate trigger. This finding suggests that, while the OVA-SEB model may more closely reflect a predominantly T2 eosinophilic CRS-like pattern, the OVA-AP model may reflect features of a mixed inflammatory phenotype. In humans, mixed endotypes have been associated with more severe clinical disease [26].

Since very few murine CRS studies use functional olfactory testing, we addressed this gap by assessing olfactory function using two complementary behavioral assays. The buried food test revealed a clear olfactory deficit in both mouse models, despite substantial inter-individual variability, whereas the habituation/dishabituation test showed no impact of eCRS on olfactory scores. This discrepancy may reflect the distinct olfactory functions assessed by each test: odor detection in the buried food test, comparable to the threshold test in humans, versus odor discrimination in the habituation/dishabituation test. Consistent with this interpretation, eCRS mice exhibited impaired odor detection, while their ability to discriminate between odors appeared largely preserved, which is consistent with findings in humans [27]. These results suggest that eCRS may differentially affect specific aspects of olfactory function, with a greater impact on odor detection than odor discrimination, which is a suprathreshold task that relies more on central neural mechanisms. Further studies will be required to determine the contributions of peripheral and central mechanisms to these alterations [28].

To investigate the epithelial changes contributing to the observed OD, we quantified the OE area in each mouse’s nasal cavity using OMP staining, a highly specific marker for mature OSNs. Measurements were taken at three levels of the skull to identify the region with the most abundant OE, which corresponded to the posterior section of the nasal cavity presenting ethmoturbinates, dorsal meatus, and septum [29]. Analysis of this posterior region in both models revealed a significant reduction in OE area in eCRS mice compared with controls in both models. Additionally, we showed structural remodeling of the OE in eCRS mice in both mouse models, characterized by a decrease in the epithelial thickness of the OE. Interestingly, this is in contrast with the typical finding of increased epithelial thickness at the level of the respiratory mucosa in eCRS [13,16,22,30,31,32]. Two other studies have reported similar findings [33,34], suggesting alternative remodeling phenomena at the level of the OE to those observed in the respiratory epithelium that need further exploration.

Interestingly, none of the individual structural or inflammatory parameters, including OSN loss, eosinophilic infiltration, or neutrophilic infiltration, showed a significant correlation with olfactory performance as measured by the buried food test. This suggests that OD in both models may not be directly proportional to the extent of neuronal loss or inflammatory infiltration and could instead result from the combined effects of multiple pathological processes. This result should be interpreted with caution, as only eight eCRS mice per model were analyzed and buried food test scores exhibited substantial inter-individual variability. In the OVA-AP 12-week mice, all animals reached the maximum time in the buried food test since they never found the food pellet, which reduced variability in olfactory performance and may have limited the ability to detect correlations with OSN loss or inflammation. While previous papers have reported a positive correlation between OSN loss and olfactory test score [16,35,36,37], control mice were also included in the analysis. Therefore, the significant global correlation likely reflects the impact of disease status rather than a direct quantitative relationship between OSN loss and olfactory dysfunction.

Some limitations of this study should be acknowledged. First, we did not compare the models across different mice strains and only BALB/c mice were used, a strain prone to strong T2 inflammatory responses. While appropriate for modeling eCRS, this choice limits generalizability to other strains, such as C57BL/6, which may exhibit different inflammatory and olfactory responses. Secondly, the study was powered for the primary olfactory outcome (buried food test), and the CTRL versus CRS comparison (n = 8 per group) provides sufficient statistical power to detect the expected effect size for this endpoint. In contrast, sex-stratified analyses, as well as secondary behavioral, histological, and correlation analyses, were performed within the same experimental cohort but were not independently powered and should therefore be considered exploratory. Accordingly, these analyses may be more sensitive to inter-individual variability and underpowered to detect weaker effects, particularly in the context of subgroup comparisons and correlation analyses. Finally, although we characterized OD and differences in inflammatory severity across models, we did not investigate the underlying cellular or molecular mechanisms; this will be within the scope of other publications.

Despite these limitations, the study provides a comprehensive comparison of eCRS mouse models, integrating histological and inflammatory assessments, as well as rarely reported measures such as sex differences and olfactory function, and highlights the models most suitable for studying OD in eCRS. A better understanding of the mechanisms underlying olfactory dysfunction may improve the characterization of this persistent symptom and support the development of more effective therapeutic strategies. These models may also serve as systems for the preclinical evaluation of targeted treatments.

## 5. Conclusions

In this study, we demonstrate that two of the most frequently reported murine models of eCRS are suitable for inducing and investigating OD. Among them, the OVA-AP 12-week model appears particularly relevant, as it induces more pronounced inflammation. These findings provide a foundation for future mechanistic studies and may support the development and preclinical evaluation of targeted therapeutic strategies for OD in eCRS.

## Data Availability

Data is contained within the article or Supplementary Material.

## Supplementary Materials

The following supporting information can be downloaded at https://www.mdpi.com/article/10.3390/cells15121118/s1. Figure S1: Post hoc analyses of two-way ANOVA with sex and group as factors. Figure S2: Comparison of olfactory and histological parameters between CTRL and eCRS mice across models. Figure S3: Association of olfactory sensory neuron loss and inflammation with olfactory function in eCRS mice.

## Abbreviations

The following abbreviations are used in this manuscript:

eCRS	Eosinophilic chronic rhinosinusitis
OD	Olfactory dysfunction
OVA	Ovalbumin
SEB	*Staphylococcus aureus* enterotoxin B
AP	*Aspergillus oryzae* protease
CRS	Chronic rhinosinusitis
CRSwNP	Chronic rhinosinusitis with nasal polyps
CRSsNP	Chronic rhinosinusitis without nasal polyps
i.p.	Intraperitoneal
i.n.	Intranasal
CTRL	Control
PBS	Phosphate-Buffered Saline
ALU	Aluminum hydroxide gel
H&E	Hematoxylin and eosin
OE	Olfactory epithelium
OSNs	Olfactory sensory neurons
SD	Standard deviation
OMP	Olfactory marker protein
