# Supplementary material for: Comparative Characterization of Olfactory Dysfunction in Mouse Models of Eosinophilic Chronic Rhinosinusitis
Source: Cells. 2026 Jun 20;15(12):1118. doi: 10.3390/cells15121118 (PMC13296451; doi:10.3390/cells15121118)
Supplement: Supplementary file 1 [file cells-15-01118-s001.zip › cells-4322776-supplementary.pdf]

Supplementary Figures

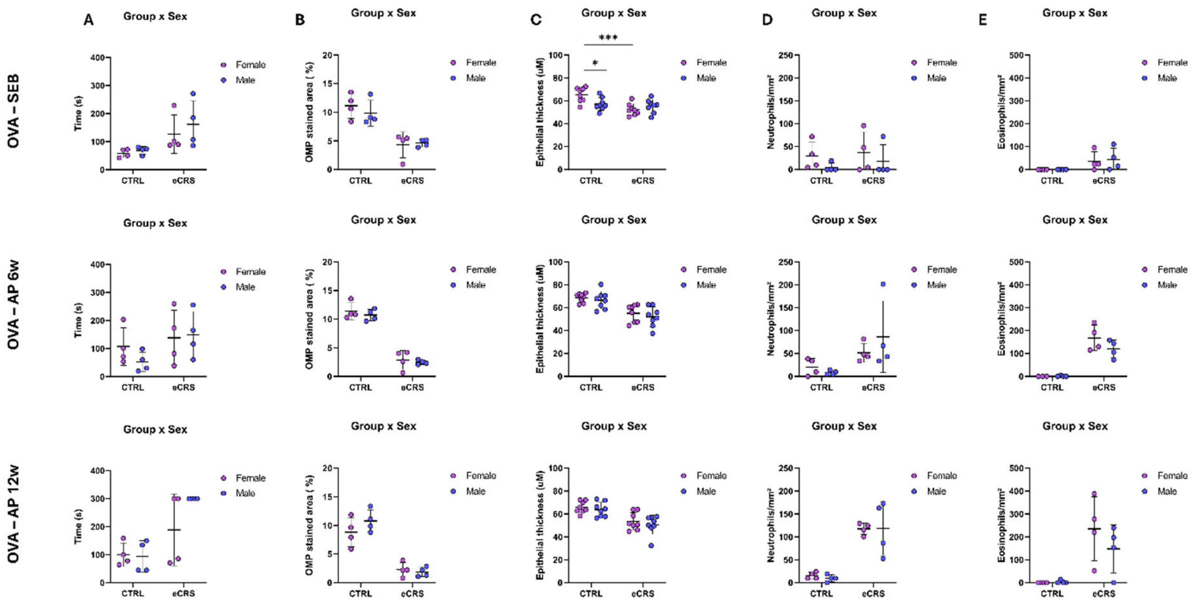

Figure 1. Post-hoc analyses of two-way ANOVA with sex and group as factor. A) Buried food test. B) Olfactory epithelial area. C) Olfactory epithelial thickness. D) Neutrophilic infiltration in the olfactory mucosa. E) Eosinophilic infiltration in the olfactory mucosa. Data are expressed as mean ± SD. Post-hoc multiple comparisons of two-way ANOVA were conducted using Sidak test: * $p < 0.05$; *** $p < 0.001$. CTRL : control; eCRS : chronic rhinosinusitis.

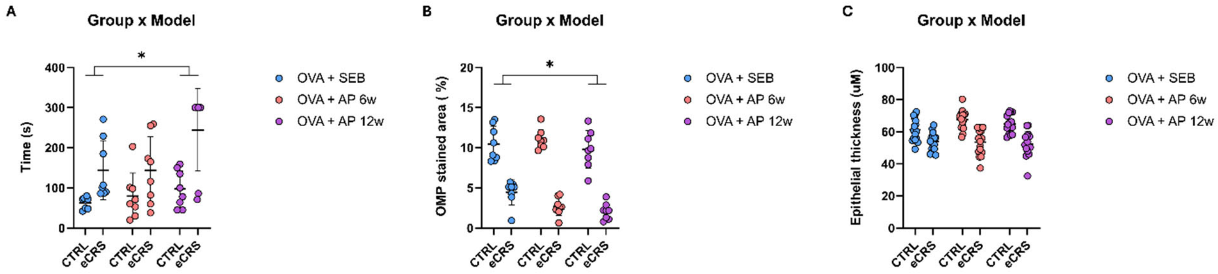

Figure 2. Comparison of olfactory and histological parameters between CTRL and eCRS mice across models. Two-way ANOVA was performed with model and group as factors. Buried food test scores (A), OMP+ stained area (B), and epithelial thickness (C) were compared between CTRL and eCRS mice across models. Data are presented as mean ± SD. Post-hoc multiple comparisons were performed using Tukey test: *$p < 0.05$. CTRL: control; eCRS: eosinophilic chronic rhinosinusitis.

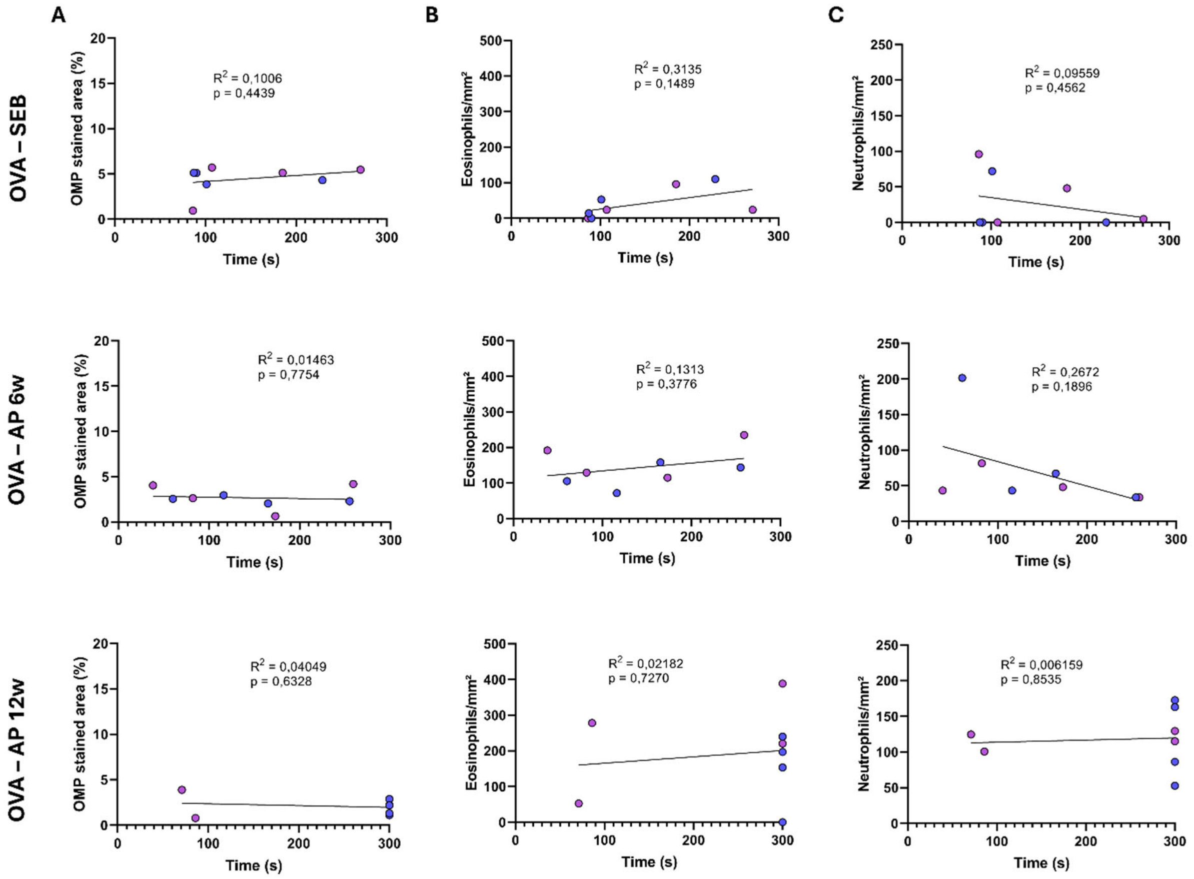

Figure 3. Association of olfactory sensory neuron loss and inflammation with olfactory function in eCRS mice. Scatter plot showing the relationship between OMP⁺ stained area (A), eosinophil counts (B), and neutrophil counts (C) and buried food test performance in eCRS mice of both mouse model. Associations were assessed using Pearson's correlation coefficient. Linear regression lines are shown for visualization. R^2 values and corresponding p-values are indicated on the graphs.
